# Entropy-Guided Regime Switching for Railway Passenger Flow Forecasting: An Adaptive EA-ARIMA-Informer Framework

**DOI:** 10.3390/e28020182

**Published:** 2026-02-05

**Authors:** Silun Tan, Xinghua Shan, Zhengzheng Wei, Shuo Zhao, Jinfei Wu

**Affiliations:** Institute of Computing Technology, China Academy of Railway Sciences Corporation Limited, Beijing 100081, China; silontan@163.com (S.T.);

**Keywords:** passenger flow forecasting, railway system, entropy-guided regime detection, adaptive forecasting framework, EA-ARIMA-Informer, interpretable forecasting

## Abstract

Railway passenger flow forecasting plays a critical role in operational efficiency and resource allocation for transportation systems. However, existing deep learning approaches suffer significant performance degradation when facing rare but high-impact events, primarily due to sample scarcity and their inability to distinguish between routine patterns and disruption regimes. To address these challenges, this study introduces EA-ARIMA-Informer, an adaptive forecasting framework that integrates entropy-augmented ARIMA with Informer through an entropy-guided regime-switching mechanism. The passenger flow series is characterized through a multi-dimensional entropy space comprising four complementary measures: Sample Entropy quantifies local regularity and predictability, Permutation Entropy captures the complexity of ordinal dynamics, Transfer Entropy measures causal information flow from external events (holidays, weather) to passenger demand, and the Conditional Entropy Growth Factor (CEGF)—a novel metric introduced herein—detects regime transitions by tracking the rate of uncertainty change between consecutive time windows. These entropy indicators serve dual roles as feature inputs for representation learning and as state identifiers for segmenting the time series into stable and fluctuating regimes with distinct predictability properties. An adaptive dual-path architecture is then designed accordingly: EA-ARIMA handles low-entropy stable regimes where linear seasonality dominates, while EA-Informer processes high-entropy fluctuating regimes requiring nonlinear residual modeling, with CEGF-guided gating dynamically controlling component weights. Unlike conventional black-box gating mechanisms, this entropy-based switching provides physically interpretable signals that explain when and why different model components dominate the forecast. The framework is validated on a large-scale dataset covering nearly 300 Chinese cities over three years (2017–2019), encompassing normal operations, holiday peaks, and extreme weather disruptions. Experimental results demonstrate that EA-ARIMA-Informer achieves a MAPE of 4.39% for large-scale cities and 7.82% for data-scarce small cities (Tier-3), substantially outperforming standalone ARIMA, XGBoost, and Informer, which yield 15.95%, 13.75%, and 12.87%, respectively, for Tier-3 cities. Ablation studies confirm that both entropy-based feature augmentation and CEGF-guided regime switching contribute significantly to these performance gains, establishing a new paradigm for interpretable and adaptive forecasting in complex transportation systems.

## 1. Introduction

Railway passenger flow forecasting confronts fundamental challenges arising from the coexistence of routine patterns and extreme disruptions within complex transportation networks. In China’s railway system, daily passenger volumes fluctuate dramatically during Spring Festival peaks, while extreme weather can suppress demand by more than 50% within hours [1]. This variability reflects the intrinsic complexity of railway systems as multi-scale adaptive networks where individual travel decisions aggregate through spatial-temporal interactions to produce emergent behaviors that defy conventional prediction approaches [2]. The operational and economic consequences of forecasting errors during these critical periods—whether holiday peaks or weather-induced disruptions—necessitate new approaches that can reliably distinguish and adapt to different operational regimes [3].

Accurate railway passenger-flow forecasting is particularly critical during rare yet high-impact disruptions, when operational decisions must be made under heightened uncertainty [4]. Deep learning has shown strong performance in time-series forecasting under normal conditions, as recurrent models (e.g., LSTM and GRU) [5,6,7] and Transformer-based architectures can capture complex temporal dependencies and, when applicable, spatio-temporal correlations. Larger-scale language-model-like [8] architectures have also been explored in related settings. However, these data-hungry models typically require abundant representative samples to learn stable patterns; in railway operations, extreme events are intrinsically sparse, and even large cities may experience only a limited number of severe disruptions over many years [9]. Consequently, deep models that work well in routine periods often generalize poorly to disruption regimes, limiting their practical value in field deployment.

This motivates hybrid forecasting frameworks that combine classical statistical models with neural networks, leveraging their complementary strengths: statistical components provide stable modeling of linear trends/seasonality with low data requirements, while neural components capture nonlinear residual structures, potentially reducing variance and predictive uncertainty [10,11,12]. Nevertheless, these methods typically rely on fixed weights or implicit gating functions, which limit their adaptability to abrupt regime changes. For instance, TCN-GRU [13] and similar hybrid architectures employ learned attention or fixed fusion strategies that cannot explicitly distinguish between stable and volatile regimes.

Information entropy provides a mathematically rigorous framework for quantifying uncertainty and detecting structural changes in complex time series. Since Shannon’s foundational work, entropy-based measures have evolved to characterize different aspects of dynamical systems: Sample Entropy quantifies the regularity and predictability of patterns, Permutation Entropy captures the complexity of ordinal dynamics, and Transfer Entropy measures directed information flow between coupled processes [14]. While these measures have been applied to analyze traffic patterns and travel demand variability, their systematic integration for state identification and adaptive model selection in railway forecasting remains unexplored [15,16].

The effectiveness of information entropy in characterizing complex dynamics has been demonstrated across diverse time series applications. In financial markets, entropy measures successfully detect regime transitions between volatile and stable periods, with Sample Entropy and Permutation Entropy providing early warning signals for market crashes [17]. Traffic flow studies reveal that entropy-based indicators can distinguish free-flow, congested, and breakdown states more reliably than traditional density or speed metrics. In power grid analysis, Transfer Entropy quantifies the risks of cascading failure by measuring information propagation between interconnected components. These successful applications share a common insight: entropy captures fundamental structural properties of system dynamics that remain invariant across different scales and contexts, suggesting potential value for railway passenger flow analysis where similar multi-regime behaviors emerge from the interaction of routine patterns, seasonal variations, and disruptive events. The theoretical advantages of entropy-based characterization align well with the challenges of forecasting railway passenger flow. Moreover, entropy’s sensitivity to distributional changes rather than absolute magnitudes could enable the detection of regime shifts even when passenger volumes vary dramatically across cities of different sizes, addressing a key limitation of volume-based forecasting approaches.

The proposed EA-ARIMA-Informer architecture leverages entropy-based state identification to address fundamental limitations in existing forecasting methods. This work makes three contributions to railway passenger flow forecasting and the broader field of transportation analytics:

- A multi-entropy characterization scheme combining sample entropy, permutation entropy, transfer entropy, and approximate entropy to quantify passenger flow complexity across multiple dimensions.
- A Conditional Entropy Growth Factor (CEGF) that enables interpretable regime detection and serves as the basis for adaptive model switching.
- An adaptive dual-path framework, EA-ARIMA-Informer, uses real-time entropy diagnostics to switch forecasting emphasis between ARIMA and Informer, capturing state-dependent linear (trend/seasonality) and nonlinear patterns while balancing accuracy and interpretability.

The remainder of this paper is organized as follows. Section 2 reviews related work on railway passenger flow forecasting methods, entropy applications in time series analysis, and hybrid forecasting architectures. Section 3 develops the theoretical foundation for entropy-based characterization of passenger flows and introduces the CEGF metric. Section 4 presents the EA-ARIMA-Informer architecture, detailing the entropy computation pipeline, dual-path design, and adaptive switching mechanism. Section 5 reports comprehensive experimental results, including comparative studies across city scales, ablation analysis, and sensitivity studies. Section 6 concludes with a discussion of practical implications for railway operations and directions for future research in entropy-guided forecasting systems.

## 2. Related Work

Railway passenger flow forecasting has evolved through distinct paradigms. Guided by the research objectives of achieving interpretability, scenario adaptability, and accuracy in railway passenger flow forecasting methods, this section synthesizes relevant research from three perspectives.

### 2.1. Evolution of Passenger Flow Forecasting Architectures from Statistical Methods to Deep Learning

The evolution of passenger flow forecasting reflects a fundamental tension between model sophistication and practical robustness. Classical statistical methods established theoretical foundations through interpretable time series decomposition. ARIMA models, as early representatives, performed well in handling passenger flow data under stationarity assumptions [18]. Dou et al. [19] identified two critical characteristics of high-speed railway passenger flow: quasi-periodic variations and complex nonlinear fluctuations, demonstrating that incorporating fuzzy logic relationship recognition could capture patterns with explicit physical meaning.

The machine learning era introduced nonlinear modeling capabilities that statistical methods inherently lacked. Support Vector Machines (SVM) achieved nonlinear prediction through kernel mappings [20], while ensemble methods such as Random Forest and GBDT further improved prediction stability [21]. A comprehensive analysis of 257 accuracy trials by Eliana Vivas et al. [22] revealed that machine learning models incorporating exogenous variables consistently outperformed classical methods under normal conditions, though this superiority depended on abundant representative training data.

Deep learning architectures pushed the computational boundaries of passenger flow prediction. Lu et al. [23] propose MulDesLSTM, a dense residual LSTM that fuses multi-time-granularity AFC passenger-flow data to improve URT forecasting accuracy, validated on Shanghai with substantial error reductions. He et al. [24] encoded multiple correlation types through MGC-RNN: spatial dependencies via network topology, temporal dependencies through LSTM structures, and latent inter-station relationships via attention mechanisms, achieving network-wide multi-step prediction. However, experiments on Shenzhen Metro data exposed a critical issue: performance degraded sharply when dealing with the scenario of prolonged passenger flow surges during the Spring Festival. Deng et al. [25] achieved 1.7–36.9% higher prediction accuracy than classical models using CEEMDAN-TCAN, but the computational overhead of two-step fuzzy k-means clustering and Shapley-based feature selection limited real-time deployment.

Recent research has shifted toward lightweight architectures to balance accuracy and efficiency. Graph neural network applications [26,27,28] demonstrated advantages in capturing spatial and long-range dependencies, enabling the model to adaptively capture evolving spatio-temporal dependencies in intelligent transportation systems. These advances indicate that architectural innovation is moving from purely pursuing accuracy toward practical considerations.

### 2.2. Information Entropy as a Mathematical Framework for Complexity Characterization

Information entropy has evolved from Shannon’s communication theory into a powerful framework for understanding complex system dynamics. Guan et al. [29] marked a paradigm shift by mapping high-order fluctuation information entropy to uncertainty membership degrees, transforming inconsistency from a modeling obstacle into a quantifiable feature. This insight resonates deeply with railway systems where passenger decisions exhibit both deterministic patterns and stochastic variations.

Theoretical foundations for complexity measurement have been established from multiple perspectives. Ponce-Flores M et al. [30] demonstrated that Spectral Entropy captures frequency-domain characteristics while Permutation Entropy identifies ordinal patterns robust to noise—complementary views essential for multi-scale passenger flow phenomena. Sample Entropy and Fuzzy Entropy excel at quantifying sequence regularity [31], while Approximate Entropy maintains stability in finite-length noisy sequences [32].

The profound contribution of entropy theory lies in establishing fundamental limits of predictability. Li et al. [33] proved that conditional differential entropy constitutes a rigorous lower bound for prediction accuracy—even state-of-the-art deep learning models can only approach, not surpass, these theoretical limits. This finding reframes our objective: identifying when and why current models operate far from theoretical limits rather than pursuing marginal architectural improvements.

Practical applications validate entropy’s versatility in capturing regime changes and structural transitions. In tourism demand forecasting [34], entropy successfully identified 6–12-month uncertainty cycles, with elasticities ranging from 1.78% to 7.21% by tourist origin. Traffic data aggregation studies [35] showed that entropy-guided temporal resolution selection could reduce forecasting errors by 14–30%.

Yet critical gaps remain in the application of entropy to railway forecasting. Existing work treats different entropy measures independently, missing potential synergies. Most importantly, no framework systematically maps entropy signatures to operational regimes in railway systems—a prerequisite for entropy-guided adaptive forecasting.

### 2.3. Hybrid Forecasting Models and Regime Identification

The theoretical foundation for hybrid forecasting rests on two key insights. First, the combination of data-driven and model-driven methods enables leveraging domain knowledge embedded in statistical formulations while exploiting the representational flexibility of neural architectures. Second, hybrid models mitigate overfitting risk by amalgamating multiple methods, thereby enhancing generalization capacity [36]. However, binary regime classification may oversimplify the continuous spectrum of conditions in railway passenger flow.

Regime identification becomes critical during pattern-disrupting events. Zhang et al. [37] addressed this through EF-former for urban rail transit, recognizing that large-scale events induce distinct operational modes requiring differentiated prediction strategies. Similarly, Yang et al. [38] proposed a Transformer-based multi-task learning method recognizing that different transportation modes exhibit distinct operational states. These multi-view perspectives inform the current study’s use of multi-dimensional entropy indicators capturing different facets of system complexity.

Recent hybrid architectures have explored various integration strategies. The IVMD-FE-Ad-Informer framework [39] introduced Fuzzy Entropy-based adaptive loss functions for regime-dependent processing. For uncertainty quantification, DeepSTUQ [40] distinguishes aleatoric and epistemic uncertainty, though treating uncertainty as output limits adaptive utility. Dynamic switching mechanisms, including TMFO-AGGRU [41], ST-HMGCN [42], and hierarchical ensembles [43], provide foundations for multi-regime handling.

Despite these advances, gaps remain in explicit regime-identification mechanisms, continuous complexity characterization, and principled regime-to-model allocation strategies.

### 2.4. Research Gaps and Contributions

Through this literature synthesis, three interconnected research gaps are identified. First, no framework systematically connects entropy signatures to operational regimes in railway systems—the relationship between different entropy types and passenger flow dynamics remains unexplored. Second, current hybrid models use fixed combination rules or black-box gating mechanisms, with entropy serving merely as an input feature rather than guiding architectural decisions. Third, existing models require extensive retraining when facing unexpected events, and the computational overhead of sophisticated methods precludes real-time response. The proposed work addresses these gaps by introducing an entropy-augmented ARIMA-Informer framework that transforms entropy from a descriptive measure to an active component in adaptive forecasting.

## 3. Entropy-Based Characterization of Railway Passenger Flow Time Series

### 3.1. Theoretical Foundation of Information Entropy

Information entropy, as a fundamental measure of system uncertainty, was first introduced by Shannon in 1948 [44]. For a discrete random variable X, Shannon entropy is defined as

(1) $$H(X)=-\sum_{i=1}^{n}p(x_{i})\log_{2}\;p(x_{i})\;$$

where $p(x_{i})$ represents the probability of outcome $x_{i}$. When applied to time series analysis, entropy effectively quantifies the complexity and predictability of sequences. In the railway passenger flow time series, entropy directly measures the randomness and predictability of the sequence.

To characterize railway passenger flow dynamics, several specialized entropy measures have been adopted: each capturing a different aspect of the signal:

**Sample Entropy (SampEn)** evaluates template-based regularity, where lower values imply strong regularity and higher values indicate complex or noisy patterns.

**Permutation Entropy (PE)** quantifies the complexity of ordinal patterns within windows, providing robustness to nonlinearities and noise.

**Transfer Entropy (TE)** measures the causal influence of exogenous events on time series.

**Conditional Entropy Growth Factor (CEGF)** is a novel indicator introduced in this work for detecting system state transitions.

These entropy measures collectively establish a signal–information perspective for railway passenger flow, elucidating the series’ current complexity and the influence of exogenous inputs on its uncertainty, providing key foundations for interpretable feature extraction and prediction.

### 3.2. An Information-Theoretic Perspective on the Railway Passenger Flow System

To establish the mapping relationship between information entropy and railway passenger flow, it is necessary to examine the railway passenger flow system from an information-theoretic perspective.

#### 3.2.1. Passenger Flow as an Information Source

The passenger flow at each time point can be viewed as a signal emitted by the system, containing information about the system state. Under normal conditions, signals exhibit regularity with lower information entropy; under abnormal conditions, signals become irregular with increased entropy. The passenger flow sequence $X=\left\{x_{1},x_{2},\ldots ,x_{n}\right\}$ constitutes an information sequence, where its probability distribution P(X) determines the information entropy H(X).

#### 3.2.2. Information Dynamics of Temporal Evolution

The evolution of the passenger flow system can be described in terms of information dynamics. Let the system state at time t be $S_{t}$, with passenger flow $x_{t}$, then the state transition can be expressed as:

(2) $$S_{t+1}=f(S_{t}\;,U_{t}\;,\epsilon_{t})$$

Where f is the state transition function, $U_{t}$ represents external inputs (weather, holidays), and $\epsilon_{t}$ is a random disturbance. The information entropy evolution of this process is:

(3) $$H(S_{t+1}|S_{t})=H(f(S_{t},U_{t},\epsilon_{t})|S_{t})=H(U_{t}|S_{t})+H(\epsilon_{t})$$

This expression indicates that the system’s conditional entropy is jointly determined by external input uncertainty and intrinsic randomness. When abnormal events occur, $H(U_{t}|S_{t})$ increases sharply, causing entropy surge.

### 3.3. Entropy Feature Extraction for Railway Passenger Flow System

To address limitations of existing railway passenger flow forecasting methods in uncertainty modeling, scenario adaptation, and interpretability, this section operationalizes four entropy-based feature families, grounded in the theoretical framework presented above.

#### 3.3.1. Entropy Feature Extraction Framework

**Figure 1:**
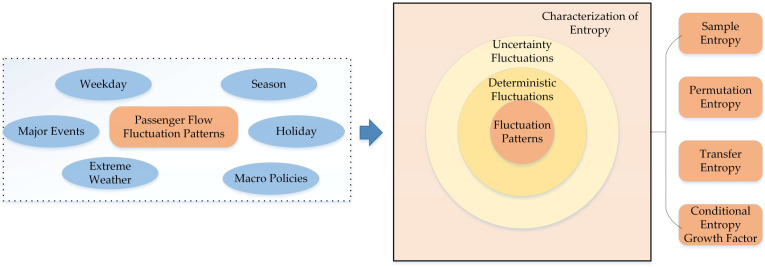
Multi-Source Drivers of Passenger Flow Fluctuations and Corresponding Entropy Feature Extraction Framework.

Figure 1 illustrates the multi-source drivers of railway passenger flow fluctuations and the corresponding entropy-based characterization. Deterministic rhythms (weekday/weekend, seasonal cycles) form the stable layer, whereas holidays, extreme weather, major events, and macro policies inject uncertainty fluctuations. Sample and Permutation Entropy capture intrinsic regularity, while Transfer Entropy and the Conditional Entropy Growth Factor characterize how exogenous events perturb the series. This unified entropy framework, therefore, underpins prediction across heterogeneous scenarios.

#### 3.3.2. Sample Entropy and Flow Regularity

Sample Entropy (**SampEn**) was proposed by Richman and Moorman in 2000 [45] to measure the regularity and complexity of time series. For a time series of length N, sample entropy is defined as:

(4) $$SampEn(m,r,N)=-ln\frac{A^{m}(r)\;}{B^{m}(r)}$$

where m is the embedding dimension (typically 2 or 3), r is the tolerance threshold (typically 0.1–0.25 times standard deviation), $A^{m}(r)$ is the number of template matches of length m and $B^{m}(r)$ is the number of template matches of length m+1.

Low **SampEn** implies stable patterns suited for short-window models; high **SampEn** calls for more sophisticated nonlinear models or additional exogenous inputs. This characteristic provides critical cues for designing stable and nonlinear prediction models.

#### 3.3.3. Permutation Entropy and Dynamic Flow Patterns

Permutation Entropy quantifies complexity by analyzing patterns of sequence arrangement [46]. For a sequence of length n, with embedding dimension m and delay τ, construct a vector:

(5) $$x_{i}=[x_{i},x_{i+\tau \;},\;\ldots ,x_{i+(m+1)\tau }]$$

Sort vector elements by magnitude to obtain permutation pattern π. Calculate the probability p(π) for all m! possible patterns:

(6) $$PE=-\sum_{\pi }p(\pi )lnp(\pi )$$

Permutation Entropy under different time-of-day and calendar effects exhibits distinct regularities. Moreover, because significant holiday periods have similar lengths, and the same holiday across different years shows comparable temporal patterns, the Permutation Entropy dynamics during holidays tend to form a relatively stable regime characteristic of each holiday type. These characteristics provide a useful basis for systematically mining the fluctuation patterns of passenger flows under holiday impacts and for refining holiday-specific forecasting strategies.

#### 3.3.4. Transfer Entropy and Event Flow Causality

Transfer Entropy [47] quantifies information transfer from event Y to flow X:

(7) $$TE_{Y\to X}\;=\sum p(x_{t+1\;},x_{t}^{\left(k\right)},y_{t}^{\left(l\right)})log\frac{p(x_{t+1}|x_{t}^{\left(k\right)},y_{t}^{\left(l\right)})}{p(x_{t+1}|x_{t}^{\left(k\right)})}$$

where $x_{t}^{\left(k\right)}$ is the *k*-dimensional history (lag vector) of passenger flow, $y_{t}^{\left(l\right)}$ is the *l*-dimensional history of the exogenous input series (e.g., holiday dummies, weather indices, or city-wide events).

By treating holidays, weather indices, or citywide events as Y, we quantify their causal influence on future passenger flow X. We can identify which types of events are the dominant causal factors for future passenger flows. This guides the construct feature windows: events with high Transfer Entropy and longer lead times should be explicitly encoded as advance warning signals.

#### 3.3.5. CEGF and Regime Detection

To capture sudden changes in flow uncertainty and detect potential regime shifts, a novel metric termed the Conditional Entropy Growth Factor (**CEGF**) is proposed. At time *t*, **CEGF** is defined as

(8) $$CEGF_{t}=\frac{H(X_{t}|X_{t-\omega :t-1})-H(X_{t-1}|X_{t-\omega -1:t-2})}{\bar{H}}$$

where $X_{t-\omega :t-1}$ is the historical flow window of length *ω* immediately preceding *t*, $X_{t-\omega -1:t-2}$ is the corresponding window one step earlier, H is the conditional entropy of the current flow given its recent history, $\bar{H}$ is a normalization constant, taken as the long-run average conditional entropy over the entire observation period, which rescales the quantity and makes **CEGF** comparable across different lines and time horizons.

From the perspective of railway passenger flows, sudden spikes in CEGF signal that the short-term flow dynamics have entered a new, more uncertain regime—for example, due to unanticipated weather disruptions, emergency incidents, or sudden policy changes.

Through Sample Entropy, Permutation Entropy, Transfer Entropy, and CEGF, the proposed framework characterizes railway passenger flow and the physical meaning of each entropy across four dimensions: regularity, dynamic patterns, causal drivers, and anomaly transitions. This multi-entropy approach directly addresses the gaps identified in Section 1 and Section 2—providing quantifiable uncertainty, scenario-aware adaptability, and interpretable pathways from external events to passenger-flow behavior, thereby underpinning the predictive modeling framework presented in the following section. Furthermore, single-entropy measures provide an incomplete characterization of complexity. A sequence may exhibit low Sample Entropy yet high Transfer Entropy. The weighted combination in CEGF enables multi-dimensional regime assessment, reducing false activations from isolated anomalies in any single indicator.

## 4. The EA-ARIMA-Informer Framework for Railway Passenger-Flow Forecasting

### 4.1. Overall Architecture of the Entropy-Guided Prediction Framework

Based on the entropy-based characterization presented in Section 3, this section introduces the core design principle of the proposed framework: positioning entropy as the “guide” rather than the “follower” of neural networks. This principle is operationalized through three interconnected mechanisms.

The first mechanism involves entropy-modulated information flow. Entropy values control information flow within the prediction network, directing the system to employ entropy-augmented deep learning components under high-entropy conditions to handle uncertainty, while activating ARIMA under low-entropy conditions, where more stable coefficients and patterns prevail. This adaptive routing ensures that each model component operates within its regime of competence.

The second mechanism concerns entropy-constrained learning. During training, entropy-extracted features are incorporated as prior knowledge, ensuring that network-learned representations conform to information-theoretic principles. By embedding entropy constraints into the learning process, the framework guides representation learning toward patterns that are both statistically significant and physically meaningful in the context of passenger flow dynamics.

The third mechanism establishes an entropy-guided decision process. Prediction strategies are determined by entropy values, which serve as interpretable signals for model selection and weight allocation. Rather than relying on opaque gating mechanisms, the framework uses entropy-based indicators—notably the Conditional Entropy Growth Factor—to make its decisions about when and how different model components contribute to the final forecast transparent.

### 4.2. Limitations of Conventional Methods

#### 4.2.1. ARIMA

ARIMA(p,d,q) models based on the Box-Jenkins methodology [48] assume time series stationarity:

(9) $$w_{t}\;={\left(1-B\right)}^{d}y_{t}$$

where B denotes the backshift operator, such that $By_{t}=y_{t-1}$, and the integer d controls the degree of differencing.

The differenced series $\left\{w_{t}\right\}\;$ is then modeled by an ARIMA(p,q) process:

(10) $$w_{t}\;=\sum_{i=1}^{p}\phi_{i}\;w_{t-i}\;+\varepsilon_{t}\;+\sum_{j=1}^{q}\theta_{i}\varepsilon_{t-j}$$

where p  and q  are the autoregressive and moving-average orders, $\left\{\phi_{i}\right\}$ and $\left\{\theta_{j}\right\}$ are AR and MA coefficients, and $\varepsilon_{t}$ is a white-noise error term with zero mean and constant variance.

Collecting all parameters in ${\hat{\Theta }}_{\mathrm{ARIMA}}=(\phi_{1},\ldots ,\phi_{p}\;,\theta_{q},\ldots ,\theta_{q}\;,\sigma^{2})$, the model is estimated by maximum likelihood:

(11) $${\hat{\Theta }}_{\mathrm{ARIMA}}=\mathrm{argmax}l(\Theta_{\mathrm{ARIMA}}|y_{1},y_{2},\;\ldots ,y_{T})$$

where l(⋅) denotes the log-likelihood of the ARIMA (p, d, q) specification. In the proposed framework, ARIMA is employed to capture the linear and relatively stable component of railway passenger-flow dynamics.

However, railway passenger flow exhibits two critical limitations under regime shifts. The first limitation concerns the violation of stationarity. During holiday peak periods, passenger flow in some cities can exceed three times the daily average—this significant surge goes far beyond the conventional fluctuation range, rendering traditional anomaly-detection thresholds based on Gaussian distribution or stationarity assumptions impractical. The second limitation involves time-varying parameters. Fixed $\left(\phi_{i},\;\theta_{j}\right)$ cannot adapt to rapid switches between weekday patterns, holidays, and sudden incidents, yielding persistent residual drifts and delayed responses.

#### 4.2.2. Informer

To model long-range temporal dependencies and nonlinear, volatile patterns, the Informer architecture is adopted, a transformer-based model tailored for long sequence forecasting [49]. Given an input sequence $X=[x_{1},x_{2},\;\ldots ,x_{L}]\in R^{L\times d_{x}}$ Informer learns a mapping to an H-step forecast:

(12) $$Y=[{\hat{y}}_{L+1},{\hat{y}}_{L+2},\;\ldots ,{\hat{y}}_{L+H}]=f_{\mathrm{Informer}}(X|\Theta )$$

where Θ denotes all learnable parameters in the encoder–decoder network.

Each encoder layer employs multi-head self-attention and a position-wise feed-forward network. For a single attention head with query, key and value matrices Q,K,V, the scaled dot-product attention is defined as:

(13) $$Attn(Q,K,V)=\mathrm{softmax}(\frac{QK^{T}}{\sqrt{d_{k}}})V$$

where $d_{k}$ is the key dimension. Multi-head attention concatenates the outputs of h heads and applies a linear projection:

(14) $$MHA(Q,K,V)=Concat({\mathrm{head}}_{1},\ldots ,{\mathrm{head}}_{h})W^{O}$$

with $W^{O}$ being a trainable projection matrix. Informer further adopts a ProbSparse variant of the above attention to keep only dominant queries, which reduces the computational complexity for long sequences.

With residual connections and layer normalization, the *l*-th encoder layer updates the hidden representations as

(15) $$H^{\left(l\right)}=\mathrm{FFN}(\mathrm{LayerNorm}(H^{\left(l-1\right)}+\mathrm{MHA}(H^{\left(l-1\right)},H^{\left(l-1\right)},H^{\left(l-1\right)})))$$

where FFN(⋅) is a position-wise feed-forward network. The decoder has a similar structure but additionally attends to the encoder outputs to generate multi-step forecasts.

(16) $$Loss_{\mathrm{MSE}}(\Theta )=\frac{1}{H}\sum_{h=1}^{H}{\left({\hat{y}}_{L+h}+y_{L+h}\right)}^{2}$$

During training, the parameters Θ are optimized by minimizing the mean squared error (MSE) between the predicted and true sequences. In our framework, Informer is used to model the nonlinear residual or fluctuating component that cannot be captured by the linear ARIMA backbone.

However, despite the Informer formulation above as a proxy, its long-term modeling capacity still faces two practical constraints. The first constraint relates to long-horizon dependence. The model’s attention mechanisms presuppose sufficient exposure to diverse long-horizon patterns; in practice, abnormal railway passenger-flow episodes (e.g., major holidays, extreme weather, sudden disruptions) are rare compared with routine days, so the model cannot fully learn stable representations of these regimes. The second constraint concerns regime imbalance. The severe class imbalance inherent in railway data means that when abnormal and normal flows are trained together, the scarce extreme-event samples are overwhelmed by normal patterns, leading to underfitted attention weights and poor generalization to unseen shocks. As a result, the Informer cannot be relied upon as a standalone solution for abnormal passenger-flow prediction.

### 4.3. The EA-ARIMA-Informer Framework

#### 4.3.1. Overall Architecture

**Figure 2:**
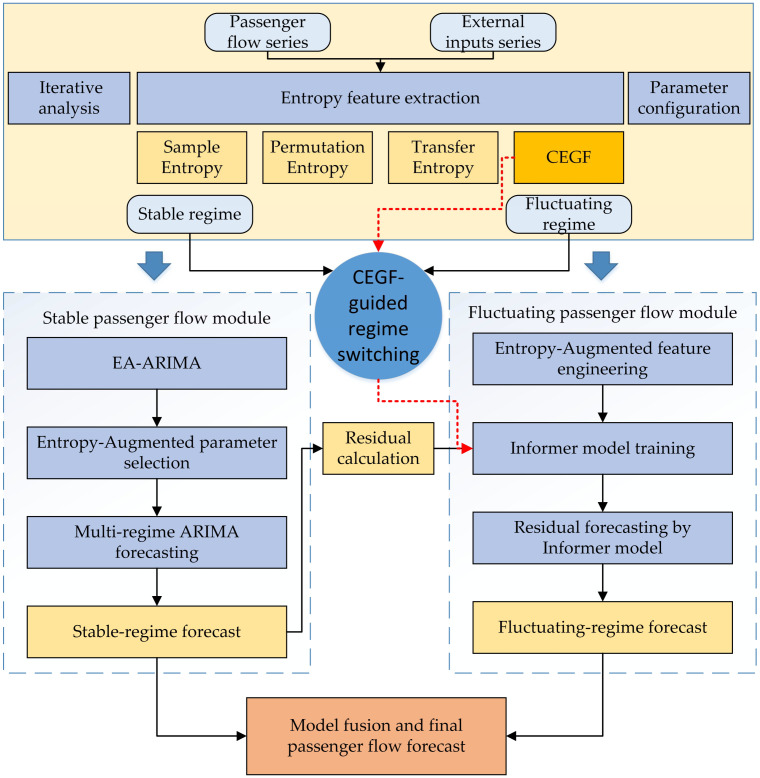
Architecture of the EA-ARIMA-Informer Framework.

The proposed EA-ARIMA-Informer framework is designed to jointly model stable and volatile railway passenger flow regimes under holiday effects and extreme events. As illustrated in Figure 2, the framework consists of three tightly coupled modules:

The first module performs entropy feature extraction. From the raw passenger-flow series and exogenous event series (holidays, weather, and large events), a set of entropy-based indicators is computed, including Sample Entropy (SampEn), Permutation Entropy (PE), Transfer Entropy (TE) and the Conditional Entropy Growth Factor (CEGF). These indicators quantify, respectively, local regularity, sequence-pattern complexity, event-flow causality and regime-shift intensity. The resulting multi-scale entropy vectors are aligned with the original time axis and serve as higher-order descriptors of flow dynamics.

The second module implements EA-ARIMA for modeling stable flows. Guided by entropy-based state segmentation, passenger flow sequences are decomposed into quasi-stationary regimes. For each regime, a dedicated ARIMA model is estimated to capture linear and seasonal structures of routine demand. This entropy-augmented ARIMA (EA-ARIMA) acts as a robust baseline for normal and mildly perturbed conditions.

The third module deploys the EA-Informer component under CEGF monitoring for modeling fluctuating flows. When CEGF signals rising uncertainty or regime shifts, a residual Informer is activated to model nonlinear, long-range deviations from the EA-ARIMA baseline. EA-Informer ingests both residual series and entropy features, and its contribution is adaptively gated by CEGF so that the system can smoothly switch between “ARIMA” and “Informer” prediction modes.

#### 4.3.2. Entropy Computation and Feature Fusion

Let $\left\{X_{t}{\}}_{t=1}^{T}\right.$ denote the univariate passenger-flow series. For each sliding window centered at time t, the following entropy measures are computed:

Sample Entropy ${\mathrm{SE}}_{t}$ quantifies local regularity and noise level. Permutation Entropy ${\mathrm{PE}}_{t}$ captures the diversity of ordinal patterns. Transfer Entropy ${\mathrm{TE}}_{t}$ measures information flow from aggregated event indices $E_{t}$ (holidays, weather, disruptions) to passenger flow $X_{t}$, reflecting exogenous driving strength. The Conditional Entropy Growth Factor ${\mathrm{CEGF}}_{t},$ as defined in Section 3.3.5, indicates the rate of uncertainty increase between consecutive windows.

These entropy values form a multi-level feature vector

(17) $$h_{t}={\left[SE_{t},PE_{t},TE_{t},CEGF_{t}\right]}^{T}$$

which is concatenated in parallel with the raw inputs and exogenous variables:

(18) $$z_{t}=[X_{t},e_{t},h_{t}]$$

Where $e_{t}$ denotes calendar, weather and events attributes.

For the EA-ARIMA module, entropy serves as a filtering and configuration signal. Time steps with persistently low **SampEn** and **PE** and small ${\mathrm{CEGF}}_{t}$ are treated as steady states, from which we estimate ARIMA parameters on relatively clean subsequences, thereby improving the identifiability of trend and seasonality.

For the EA-Informer module, entropy is embedded as an explicit feature channel, enhancing the model input after entropy channel normalization. On the other hand, CEGF serves as the core-guided mechanism, which reduces noise in Informer training and enhances the capability to capture nonlinear patterns in complex, volatile scenarios.

#### 4.3.3. State-Dependent Model Switching with Entropy Guidance

To explicitly handle heterogeneous flow regimes, an entropy-driven, state-dependent forecasting mechanism is introduced.

The first step involves state segmentation via **SampEn** and **CEGF**. Using sliding-window **SampEn** and **CEGF**, each time step is assigned to one of two macro-states consistent with the framework diagram. The stable regime corresponds to normal operating conditions. Low **SampEn** and low **CEGF** indicate regular, predictable dynamics dominated by routine commuter demand. The fluctuating regime corresponds to abnormal conditions. Elevated **SampEn** or **CEGF** signals abnormal volatility, including both sharp increases and sharp decreases in flow, typically induced by holidays, extreme weather, or sudden service disruptions. These binary state labels are used both to select the appropriate forecasting module and as auxiliary inputs.

The second step implements multi-state EA-ARIMA. Compared with a single global ARIMA, the proposed EA-ARIMA enhances linear forecasting by learning state-specific ARIMA models in statistically more homogeneous subsets and dynamically activating the appropriate parameter set according to the monitored operating state. The ARIMA parameters (p, d, q) for both high-entropy and low-entropy regimes are determined through a systematic identification procedure using the Akaike Information Criterion (AIC) and Bayesian Information Criterion (BIC). In the dual-regime configuration, parameters are estimated separately on training subsets corresponding to each entropy state, allowing regime-specific dynamics to be captured. For each regime s∈{stable, fluctuating}, a separate ARIMA ${\left(p,\;d,\;q\right)}^{\left(s\right)}$ model is estimated using only samples from that state. During inference, the current entropy profile determines the active state ${\hat{s}}_{t}$, and the corresponding ARIMA parameters are chosen to produce the baseline forecast ${\hat{X}}_{t}^{\mathrm{EA}-\mathrm{ARIMA}}$. Thus, the stable regime employs parsimonious ARIMA structures, while the fluctuating regime can invoke richer autoregressive forms and shorter rolling windows.

The third step applies the residual Informer with entropy-controlled fusion. On top of EA-ARIMA, the Informer learns the nonlinear residual process:

(19) $$R_{t}=X_{t}-X_{t}^{EA-ARIMA}$$

The encoder takes as input historical residuals $\left\{R_{\tau }{\}}_{\tau \leq t}\right.$, entropy features $\left\{h_{\tau }{\}}_{\tau \leq t}\right.$, and regime labels, so that attention weights, temporal encodings, and gating behavior are all conditioned on entropy-driven regime information. The final forecast is obtained via a CEGF-based gating mechanism:

(20) $${\hat{X}}_{t+1}=w_{t+1}({\hat{X}}_{t+1}^{EA-ARIMA}+{\hat{R}}_{t+1})+(1-w_{t+1}){\hat{X}}_{t+1}^{EA-ARIMA},w_{t+1}=\sigma (\alpha \cdot CEGF_{t+1})$$

where σ(⋅) is the sigmoid function and α is a learnable coefficient. This simple yet learnable gating function makes the fusion weight an explicit, monotonic function of the detected volatility level, providing a transparent link between the monitored state and the contribution of the residual Informer. As can be seen from Equation (20), the gating function imposes a monotonic mapping between the conditional entropy-guided factor (CEGF) and the weight assigned to the nonlinear component. The high entropy does not necessarily imply nonlinear dynamics; nevertheless, it captures most practical variations (e.g., major conferences, concerts, and festivals). Meanwhile, a small subset of disturbances may increase entropy while still being well approximated by a linear model.

In training, we jointly adjust Informer parameters, residual mapping, and the gating coefficient α, so that the model learns when and how strongly to rely on entropy-Augmented residuals. Rising entropy in the fluctuating regime pushes $w_{t+1}\to 1$, amplifying the Informer’s contribution, while low entropy in the stable regime drives $w_{t+1}\to 0$, reverting to the ARIMA base for robustness.

#### 4.3.4. Evaluation Metrics

For quantitative evaluation, both mean absolute percentage error (MAPE) and root mean squared error (RMSE) are reported.

(21) $$\mathrm{MAPE}=\frac{100\%}{N}\sum_{t=1}^{N}\left|\frac{{\hat{y}}_{t}-y_{t}}{y_{t}}\right|$$

(22) $$\mathrm{RMSE}=\sqrt{\sum_{t=1}^{N}\left({\hat{y}}_{t}-y_{t}\right)}$$

where $y_{t}$ and ${\hat{y}}_{t}$ denote the observed and predicted passenger flows at time t, and N is the number of forecast points. Lower MAPE and RMSE indicate better forecasting performance. It is noted that MAPE becomes undefined when actual values equal zero. However, for railway passenger flow forecasting at the city level, daily dispatched volumes are invariably positive due to continuous operational schedules.

## 5. Experiments and Results

### 5.1. Data Description

The proposed EA-ARIMA-Informer framework is evaluated on city-level railway passenger flow data from China. The dataset records daily departures and arrivals for nearly 300 major cities in China, where each observation represents the total number of passengers who use railways as the travel mode between a departure city and an arrival city on a given day (both outbound and inbound flows are included). The time span covers 2017–2019, yielding approximately 1.6 million city–date samples after data cleaning.

To capture calendar-driven demand shifts, a national holiday dictionary is constructed. It enumerates all statutory public holidays in China, including the start and end dates, the number of vacation days, and the holiday name (e.g., Qingming Festival, Labor Day, National Day, Dragon Boat Festival, Mid-Autumn Festival, New Year’s Day, Summer Vacation and Spring Festival). Table 1 summarizes the typical vacation duration for each major holiday. Summer vacation is not a statutory public holiday in China; however, due to school breaks for students and the common practice of taking annual leave among working adults, travel demand typically increases substantially during this period.

Over the three-year observation period, the dataset covers a total of 374 holiday-affected days. Among these, National Day holidays account for 34 days, Spring Festival travel and Summer Vacation together contribute 244 days, and other public holidays account for the remaining 96 days.

To examine the impact of extreme weather on passenger flows, a national daily weather dataset is integrated, and an extreme-weather catalog is constructed. Events are categorized into several types—including typhoon, earthquake, blizzard, moderate-to-heavy snow, heavy rain, freezing rain, and strong winds—and each type is assigned an impact level based on historical disruption severity. The dataset contains 3047 city-day instances affected by severe conditions. Specifically, 1176 instances are associated with heavy rainfall, 1596 with typhoons, and 275 with other types of extreme weather. The classification scheme is presented in Table 2.

Holiday indicators are deterministic and known in advance. Severe weather forecast errors are generally minor for short-term horizons and are treated as negligible in this study. In this set of experiments, all models are evaluated under a one-step-ahead forecasting setting, which reflects routine field practice where operators review the latest next-day passenger-flow forecast during morning-shift handover meetings to support operational decision-making. The model uses a fixed-length rolling window: when a new day’s data become available, they are incorporated into the input by shifting the window forward, so the latest observation is included while the window length remains unchanged.

All experiments are implemented on a distributed computing platform. Data storage and preprocessing are performed using Hadoop 3.3.4 and Spark 3.4.1 stack, while feature engineering, model training, and forecasting are implemented in Python 3.8, leveraging libraries, including stats 0.13.5, scikit-learn 1.2.2, torch 2.0.1, and darts 0.26.0.

### 5.2. Entropy-Based Characteristics of Railway Passenger Flows

This subsection analyzes how different entropy measures—Sample Entropy (**SampEn**), Permutation Entropy (**PE**), Transfer Entropy (**TE**), and the Conditional Entropy Growth Factor (**CEGF**)—capture the fluctuation patterns of railway passenger flows. These analyses provide quantitative evidence that entropy indicators not only describe complexity but also align closely with operational regimes, thereby justifying their use in the EA-ARIMA-Informer framework.

#### 5.2.1. Sample Entropy and Overall Predictability

**Figure 3:**
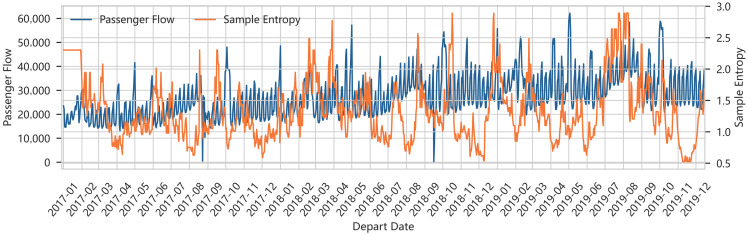
Time Series of Sample Entropy for City-Level Daily Passenger Flows.

Figure 3 plots the time series of sample entropy for three consecutive years. Across most dates, SampEn remains below 2, and it never exceeds 3, indicating that the short-term dynamics of aggregated passenger flows exhibit moderate regularity rather than pure randomness.

Several distinct patterns emerge. Workday baselines form low-entropy plateaus, reflecting highly repeatable commuting demand. Holiday periods cause temporary bumps in SampEn, but the increases are modest—the rise-and-fall trajectories within each holiday window are themselves regular, preventing entropy from exploding. Extreme-event episodes correspond to localized spikes, but the spikes are short-lived. The bounded SampEn profile confirms that most city-level flows remain predictable, underpinning the design of EA-ARIMA as a low-entropy baseline.

#### 5.2.2. Permutation Entropy and Holiday Pattern Consistency

**Figure 4:**
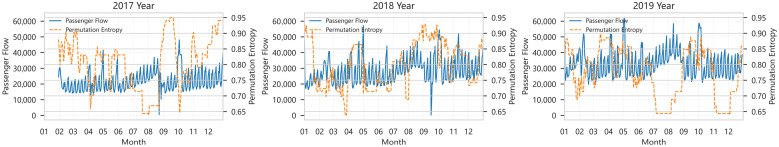
Permutation Entropy Trajectories Across Three Years Highlighting Recurrent Holiday Patterns.

Figure 4 presents permutation entropy (PE) trajectories for the same three-year period. Compared with SampEn, PE is more sensitive to the ordering of local ups and downs in the passenger-flow series.

Major holidays with fixed calendar positions—such as National Day and Spring Festival—exhibit highly similar PE profiles across different years. Non-holiday intervals show relatively flat, low permutation entropy, confirming that ordinary demand fluctuations follow similar day-of-week cycles year after year. This cross-year consistency demonstrates that holiday-induced fluctuations belong to a recurrent dynamic regime, enabling the model to leverage historical holiday patterns from previous years.

#### 5.2.3. Transfer Entropy and Event Disturbances

**Figure 5:**
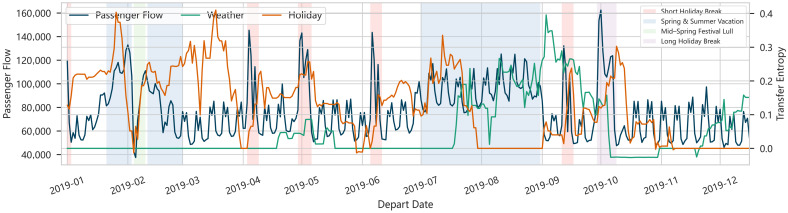
Transfer Entropy from External Event Processes to Passenger Flows.

Figure 5 depicts the transfer entropy from various external event processes (holidays, extreme weather) to passenger flows, where dark-shaded bands indicate calendar-defined holiday periods. TE measures the directed information flow from events to future passenger volumes, beyond what is already explained by the historical flows themselves.

Two primary patterns are observed. Around major holidays, TE spikes sharply in tandem with positive flow deviations. During severe typhoons, heavy rains, or earthquakes, TE also exhibits pronounced peaks, but with volumes declining substantially below normal baselines. Thus, TE captures both upward and downward disturbances as long as the event contains predictive information. The alignment between TE spikes and event windows confirms that transfer entropy effectively detects causally influential events.

#### 5.2.4. Conditional Entropy Growth Factor and Regime Detection

**Figure 6:**
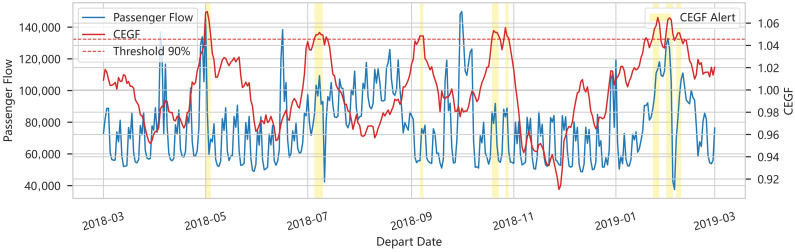
Conditional Entropy Growth Factor (CEGF) Time Series with Identified High-Volatility Regimes.

Figure 6 shows the Conditional Entropy Growth Factor (CEGF) over time, with dark-shaded regions denoting intervals where CEGF exceeds a monitoring threshold. CEGF measures the relative increase in conditional entropy between consecutive windows, capturing abrupt changes in predictability.

Periods flagged by high CEGF correspond to sharp regime transitions, including the onset of holiday peaks and sudden drops during extreme weather. Conversely, low-CEGF segments align with routine operations where daily flows are well explained by simple autoregressive structures. These observations validate CEGF as an effective regime-shift detector, directly supporting the CEGF-driven gating mechanism in the EA-ARIMA-Informer algorithm.

### 5.3. Validation of the EA-ARIMA-Informer Framework

#### 5.3.1. Experimental Design

To rigorously evaluate the proposed framework, experiments are conducted on data from June 2019 onward, a period that includes multiple holiday peaks and several extreme-weather events. Specifically, the training set is constructed using data from 2017 to 2018, while the validation set is derived from data collected between January and May 2019. In addition, cities are stratified into four categories on the basis of average passenger volume and network importance, namely Large-scale cities, Tier-1, Tier-2, and Tier-3. Cities are categorized following China’s official urban hierarchy based on comprehensive indicators, including GDP, population, and transportation infrastructure.

The experimental design incorporates three key dimensions. First, multi-scenario coverage is ensured by separately analyzing forecasting performance on workdays, holidays, and extreme weather days to test robustness across regimes. Second, a multi-method comparison is conducted by comparing EA-ARIMA-Informer against representative baselines from traditional time series (ARIMA), machine learning (XGBoost), and deep learning (Informer), as well as intermediate entropy-enhanced variants (EA-ARIMA and EA-Informer). Third, ablation studies are performed to dissect the contribution of entropy features and the CEGF-based switching. Ablation variants include ARIMA-Informer, Feature-ARIMA-Informer, and the full EA-ARIMA-Informer.

Forecast accuracy is assessed by using Mean Absolute Percentage Error (MAPE) and Root Mean Squared Error (RMSE), computed over rolling-origin forecasts with realistic look-ahead horizons.

#### 5.3.2. Training Configuration

Table 3 summarizes the hyperparameters for entropy calculation, ARIMA modeling, and Informer architecture. All configurations remain constant across experiments to ensure reproducibility and fair comparison.

For EA-ARIMA, two separate models are trained: the low-entropy model captures stable weekday dynamics with a constant trend term, while the high-entropy model accommodates the irregular patterns during festivals and extreme weather. The 90th percentile threshold for CEGF is calibrated to align with operational regime boundaries identified by railway authorities, covering pre-holiday and post-holiday transitional periods.

#### 5.3.3. Overall Forecasting Performance

**Figure 7:**
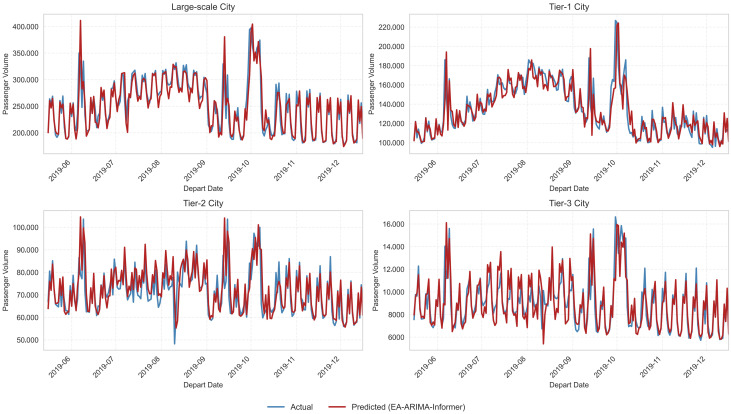
Forecasting Results across City Levels and Operational Regimes.

Figure 7 plots actual and predicted flows for representative cities. Visually, the EA-ARIMA-Informer curves track both the baseline levels and sudden peaks/drops with high fidelity, including the rise and fall around holidays and the sharp reductions during extreme weather. This confirms that the entropy-guided dual-path architecture captures both stable trends and complex nonlinear deviations.

Table 4 summarizes the average MAPE of the proposed EA-ARIMA-Informer model under different city levels and scenarios (workdays, holidays, extreme weather). Several insights emerge from these results.

First, high accuracy is achieved across all city levels. Even for smaller cities with more irregular demand and lower passenger volumes, the MAPE remains below 8%, which is sufficient for operational planning.

Second, scenario-dependent difficulty is evident. Across all city levels, workday forecasts are the most accurate (MAPE ranging from 3–7%), while holiday and extreme-weather scenarios are more challenging. This is expected: holidays introduce sustained high-volume peaks, and extreme weather yields abrupt drops and partial cancellations.

Third, a city-size effect is observed. Larger cities exhibit lower MAPE than smaller ones. The consistent performance gradient across city levels illustrates that the proposed framework adapts gracefully to data-scarce, noisy environments while taking full advantage of regularities in major hubs.

#### 5.3.4. Comparison with Benchmark Methods

**Figure 8:**
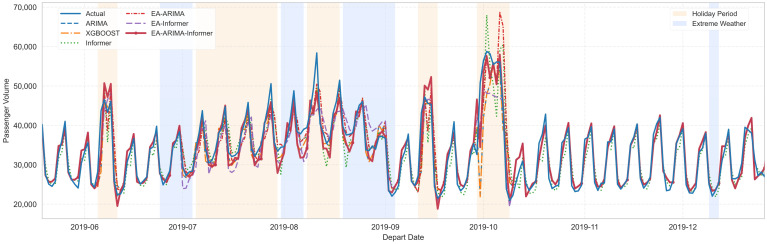
Multi-Method Forecasting Comparison Across Operational Regimes.

Figure 8 further illustrates that EA-ARIMA-Informer tracks three types of segments—workdays (white), holiday surges, and extreme weather drops—with consistently smaller deviation than all baselines. The superiority is especially notable during transition phases, where the CEGF-driven switching quickly reallocates weight from ARIMA to Informer. The comparison includes six methods: ARIMA, XGBOOST, Informer, EA-ARIMA, EA-Informer, EA-ARIMA-Informer. To ensure fair comparison, the hyperparameters of all models are tuned via grid search with validation splits.

Several consistent patterns can be observed from Table 5, the bold values denote the forecasting performance of EA-ARIMA-Informer, which achieves the best results across the reported metrics.

First, conventional models struggle in volatile regimes. ARIMA yields the worst performance across all city levels (e.g., 9.39% MAPE for large-scale cities, 15.95% for Tier-3), reflecting its limited ability to handle nonlinearities and irregular shocks.

Second, machine learning and deep learning offer partial improvements. XGBoost and vanilla Informer improve upon ARIMA, but the gains are modest, resulting in imprecise predictions during extreme events.

Third, entropy-aware single-path enhancements help but are not sufficient. EA-ARIMA and EA-Informer both outperform their non-entropy counterparts, indicating that entropy-based state segmentation and features already add value. However, each method alone still faces trade-offs: EA-ARIMA remains less flexible in highly nonlinear regimes, whereas EA-Informer is more data-hungry and less robust in stable regimes.

Fourth, the full EA-ARIMA-Informer delivers the best and most consistent performance. The proposed dual-path framework achieves the lowest MAPE and RMSE at every city level. For large-scale cities, MAPE is reduced to 4.39%, and RMSE to 20,928, both significantly better than any single model. For Tier-3 cities, the improvement is even more pronounced (MAPE of 7.82% vs. 12.87–15.95% for deep learning and conventional baselines). The small sample of Tier-1 cities limits the strength of category-level generalization claims. Nonetheless, consistent improvements across all Tier-3 cities suggest that the entropy-based regime identification captures shared operational dynamics rather than city-specific artifacts. Further validation on additional metropolitan networks would strengthen the evidence for generalizability.

These gains confirm that leveraging ARIMA for stable regimes and Informer for fluctuating regimes, under entropy guidance, is more effective than uniformly applying any single model.

#### 5.3.5. Role of Entropy and Switching Mechanism

**Figure 9:**
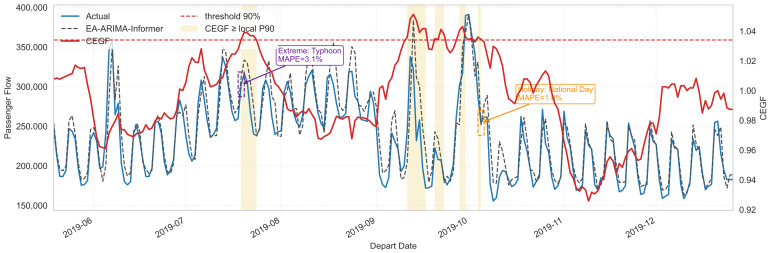
Impact of CEGF-Driven Switching on External Event Forecasting.

Figure 9 focuses on a large-scale city with daily flows between 20 k and 40 k, showcasing segments that include both sharp increases and sharp decreases. The CEGF-driven mechanism identifies when the system enters or exits abnormal regimes (e.g., holiday surges, extreme weather drops).

In holiday buildup and release periods, CEGF rises as conditional entropy grows. The gating weight, correspondingly, increases, giving more authority to the Informer residual path. The combined forecast accurately tracks the steep slopes and peak magnitudes, whereas EA-ARIMA alone tends to underestimate peaks and over-smooth turning points.

During extreme-weather-induced drops, CEGF also spikes, marking a regime transition into an abnormal fluctuating state. Informer, conditioned on entropy and external event features, learns the magnitude of residual corrections required to adjust the ARIMA baseline downward. As a result, the combined forecast captures the depth better than either ARIMA or Informer alone.

#### 5.3.6. Ablation Study

To disentangle the contributions of entropy features and CEGF-based switching, ablation experiments are conducted under holiday plus or extreme-weather settings, comparing three model variants. All variants maintain identical training strategies, feature sets, and data partitioning. The only difference lies in the fusion mechanism:

(1) ARIMA-Informer, which represents a direct hybrid without entropy. In this configuration, ARIMA and Informer are jointly trained but without explicit regime identification.
(2) Feature-ARIMA-Informer, where entropy features are added to the inputs, but no CEGF-driven switching is applied. This variant relies solely on feature augmentation.
(3) EA-ARIMA-Informer (full), in which both entropy feature extraction and CEGF-based state recognition and gating are enabled.

**Figure 10:**
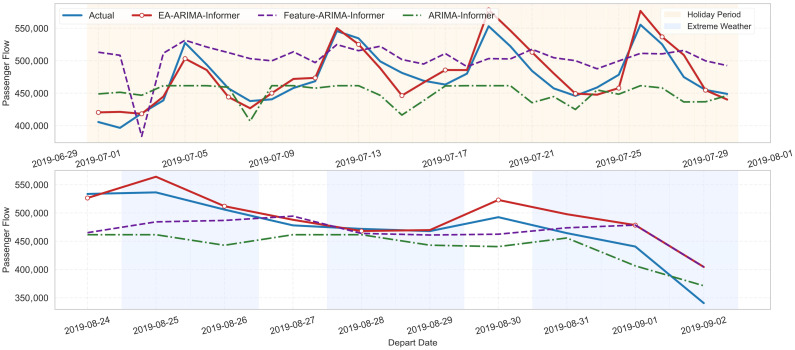
Ablation Analysis Forecasting Performance under Holiday and Extreme-Weather Scenarios.

Figure 10 corroborates these findings visually. During the summer transportation peak, where demand transitions from regular periods to sustained high-volume holiday flows, the model without CEGF tends to lag in recognizing the new regime. In contrast, EA-ARIMA-Informer, empowered by entropy-based detection, adjusts earlier and more sharply, producing predictions that align more closely with the observed surge.

Table 6 shows that moving from ARIMA-Informer to Feature-ARIMA-Informer yields some improvement, confirming that entropy features alone enhance the representation of fluctuations. Furthermore, the full EA-ARIMA-Informer delivers the largest improvements across most city levels (e.g., MAPE in large-scale cities drops further to 4.64%, and in Tier-2 cities from 11.43% to 8.33%). This highlights the crucial role of CEGF-based regime detection and model switching.

In particular, for Tier-3 cities, where data are noisy and event samples are rare, the complete Entropy-Augmented framework illustrates that entropy-guided structure is especially valuable when pure data-driven learning is fragile.

In summary, the ablation study confirms that the combination of entropy feature extraction and CEGF-based state switching is essential. Feature augmentation alone cannot fully uncover the latent structure of abnormal regimes; conversely, a switching mechanism without well-designed entropy indicators lacks reliable triggers. Only their integration yields the substantial performance and interpretability gains observed in the experiments.

### 5.4. Discussion

The experimental results yield several key insights regarding the role of entropy and the effectiveness of the EA-ARIMA-Informer framework. These findings not only validate the proposed methodology but also reveal fundamental characteristics of railway passenger flow dynamics that have implications for both theoretical understanding and practical deployment.

#### 5.4.1. Entropy as an Interpretable Lens for Passenger Flow Complexity

The entropy-based characterization presented in Section 5.2 reveals that railway passenger flows possess inherent structural properties amenable to information-theoretic analysis. As shown in Figure 3, Sample Entropy remains consistently below 3 throughout the three-year observation period, indicating that even during significant disruptions, the system never approaches purely random behavior. This bounded complexity profile has profound implications for model design: it suggests that ARIMA-type models retain fundamental value for capturing baseline dynamics, and that the forecasting challenge lies not in modeling randomness but in detecting and adapting to regime transitions. This finding directly informed the dual-path architecture of EA-ARIMA-Informer, where ARIMA handles low-entropy stable periods rather than being replaced entirely by deep learning components.

The cross-year consistency of Permutation Entropy patterns during holidays, as illustrated in Figure 4, provides empirical justification for leveraging historical holiday data in forecasting models. The observation that National Day and Spring Festival exhibit highly similar PE trajectories across 2017, 2018, and 2019 demonstrates that holiday-induced fluctuations follow recurrent dynamic templates rather than exhibiting year-specific idiosyncrasies. This regularity enables the framework to treat holiday forecasting as a pattern-matching problem augmented by entropy-guided adaptation, rather than requiring the model to learn each holiday’s dynamics from scratch with limited samples.

#### 5.4.2. Asymmetric Challenges of Holiday Surges Versus Extreme Weather Disruptions

A nuanced finding emerges from comparing forecast performance across different scenarios in Table 4. For large-scale cities, holiday forecasting proves more challenging than extreme weather prediction (MAPE of 6.33% versus 4.11%), whereas for Tier-3 cities, this pattern reverses with extreme weather presenting greater difficulty (8.22% versus 9.76% for holidays, though both remain challenging). This asymmetry reflects fundamentally different mechanisms underlying these two types of disruptions. Holiday surges involve systematic demand amplification where passenger volumes increase predictably in magnitude but with complex timing dynamics around pre-holiday departures and post-holiday returns. Large cities, serving as major origin-destination hubs, experience these surges most intensely, making accurate peak magnitude estimation difficult even with entropy guidance.

In contrast, extreme weather events induce demand suppression with relatively predictable patterns once the event is detected—passengers either cancel or postpone travel, leading to flow reductions that, while dramatic, follow more consistent trajectories than holiday-induced surges. The Transfer Entropy analysis in Figure 5 corroborates this interpretation: TE spikes symmetrically for both upward and downward disturbances, but the information content differs qualitatively. Weather-induced TE spikes tend to be sharper and more localized, enabling faster model adaptation through the CEGF mechanism, whereas holiday-induced TE elevations are more diffuse and sustained, requiring longer adaptation windows.

However, the proposed framework does not fully cover all disruption scenarios. It shows partial adaptability to localized demand surges caused by special events (e.g., football matches, concerts, exhibitions), which are typically short-lived and station-specific and thus appear as local entropy increases; in such cases, the CEGF module can flag anomalies and shift emphasis toward EA-Informer, but performance depends on whether comparable event signatures exist in the training history (recurring events at fixed venues are more learnable than unprecedented ones). In contrast, the framework is not well-suited to demand-decrease scenarios such as public health emergencies (e.g., COVID-19), because these events produce sustained structural breaks—persistent demand suppression and long-term changes in travel behavior—rather than a transient entropy spike followed by recovery, which is the regime the method is designed for.

#### 5.4.3. Synergistic Benefits of the Hybrid Architecture and Regime Switching

The benchmark comparison in Table 5 reveals that EA-ARIMA-Informer achieves performance superior to any single-component model, and importantly, this superiority represents genuine synergy rather than simple averaging. For Tier-3 cities, where the performance gap between EA-ARIMA-Informer (7.82%) and the better single-path variant EA-Informer (9.71%) reaches 1.89 percentage points—a relative improvement of approximately 19%. The magnitude of this improvement in data-scarce environments underscores a central thesis of this work: entropy-based regime identification transforms the forecasting challenge from a data quantity problem to a state detection problem. When training samples for extreme events are inherently limited, as they are for smaller cities experiencing fewer disruptions, the ability to recognize regime shifts through entropy signatures becomes more valuable than attempting to learn event-specific patterns directly from insufficient data.

The ablation study in Table 6 disentangles the contributions of entropy features and CEGF-based switching, revealing that both components are necessary but neither alone is sufficient. Moving from ARIMA-Informer (no entropy) to Feature-ARIMA-Informer (entropy features without switching) reduces MAPE for large-scale cities from 6.41% to 5.79%—an improvement of 0.62 percentage points attributable to enhanced input representation. However, the subsequent improvement from Feature-ARIMA-Informer to full EA-ARIMA-Informer (5.79% to 4.64%) contributes an additional 1.15 percentage points, nearly twice the gain from features alone. This demonstrates that entropy features, while valuable, cannot substitute for explicit regime-aware model switching.

A limitation of the current design is that the sigmoid gate enforces a monotonic mapping between CEGF and the weight; higher entropy complexity implies greater reliance on deep modeling, but potentially restrictive. In particular, some persistent interventions (e.g., substantial fare changes) may shift the passenger-flow level while remaining approximately linear in their dynamics, in which case a strictly monotonic increase in deep-model weight with respect to entropy may be suboptimal.

#### 5.4.4. Implications for Operational Deployment

Beyond accuracy improvements, the Entropy-Guided framework offers practical advantages for railway operations that merit discussion. The interpretability provided by entropy indicators addresses a significant limitation of pure deep learning approaches. As demonstrated in Figure 9, the CEGF signal provides transparent justification for model behavior: when CEGF exceeds the monitoring threshold, operators can understand that the system has detected elevated uncertainty warranting increased reliance on the Informer component. This interpretability facilitates human oversight and enables operators to validate or override model decisions based on domain knowledge—a capability absent in conventional black-box ensemble methods.

The computational efficiency of entropy computation also supports real-time deployment. In a typical deployment scenario, passenger-flow data are aggregated on a daily basis, and the forecasting system updates its predictions in the early morning before service begins (e.g., between 00:00 and 05:00). The entropy metric is computed from a sliding window over historical observations, with a time complexity between $O(N^{2})$ and O(N log N), where N denotes the window length, which meets the runtime requirements for field deployment.

## 6. Conclusions

Railway passenger flow forecasting presents a fundamental challenge arising from the coexistence of predictable routine patterns and rare but high-impact disruptions. Conventional deep learning approaches, despite their success under normal conditions, suffer significant performance degradation during extreme events due to inherent sample scarcity and the inability to distinguish between stable and volatile operational regimes. This study addressed these limitations by proposing EA-ARIMA-Informer, an adaptive forecasting framework that transforms entropy from a passive descriptive statistic into an active structural element guiding model behavior.

The framework introduces a multi-dimensional entropy characterization of passenger flow dynamics through four complementary measures. Sample Entropy quantifies local regularity and predictability, Permutation Entropy captures the complexity of ordinal patterns, and Transfer Entropy measures causal information flow from external events to passenger demand. Most notably, a novel metric—the Conditional Entropy Growth Factor (CEGF)—is proposed to detect regime transitions by tracking the rate of uncertainty change between consecutive time windows. These entropy indicators serve dual roles: as enhanced features for representation learning and as interpretable signals for adaptive model selection.

The dual-path architecture leverages entropy-based state identification to allocate forecasting responsibility between complementary components. EA-ARIMA handles low-entropy stable regimes where linear seasonality dominates, while the Informer module processes high-entropy volatile periods requiring nonlinear residual modeling. The CEGF-Guided gating mechanism dynamically controls component weights, enabling smooth transitions between ARIMA-dominant and Informer-augmented prediction modes. Unlike conventional black-box gating mechanisms, this entropy-based switching provides physically interpretable signals that transparently explain when and why different model components dominate the forecast.

Comprehensive experiments on a large-scale dataset covering nearly 300 Chinese cities over three years (2017–2019) validate the effectiveness of the proposed framework. EA-ARIMA-Informer achieves an MAPE of 4.39% for large-scale cities and maintains accuracy below 8% even for data-scarce small cities, substantially outperforming standalone ARIMA (15.95%), XGBoost (13.75%), and Informer (12.87%). The ablation study reveals that entropy-based feature augmentation contributes 0.62 percentage points improvement in MAPE, while CEGF-Guided regime switching contributes an additional 1.15 percentage points—demonstrating that explicit regime detection provides nearly twice the benefit of feature enhancement alone. The synergistic combination of both components yields performance superior to any single-path variant, with the largest relative improvements observed in data-scarce environments where entropy-driven structure compensates for limited training samples.

Several key insights emerge from this research that extend beyond the specific application domain. First, the bounded entropy profile observed across three years of passenger flow data—with Sample Entropy consistently below three even during major disruptions—demonstrates that complex transportation systems retain inherent predictability that classical time-series models can exploit. This finding challenges the assumption that deep learning should wholesale replace statistical methods for transportation forecasting. Second, the cross-year consistency of Permutation Entropy patterns during holidays reveals that extreme events, while rare, exhibit recurrent dynamic signatures amenable to entropy-based detection. This transforms the forecasting challenge from a data quantity problem to a state detection problem, enabling effective predictions even with limited historical examples of disruptions. Third, the asymmetric performance patterns across scenarios and city scales highlight the need for adaptive frameworks that can tailor their behavior to local characteristics rather than applying uniform approaches.

From a practical perspective, the entropy-guided framework offers advantages beyond accuracy improvements. The interpretability provided by entropy indicators enables human oversight of automated forecasting systems, as operators can observe CEGF signals to understand when and why the model shifts between components. The computational efficiency of entropy measures supports real-time deployment without the latency associated with model retraining, making the framework suitable for operational decision-making during rapidly evolving situations.

The methodology developed in this study establishes a transferable paradigm applicable beyond railway systems. The principle of using information-theoretic measures to guide adaptive model selection between classical and deep learning components can be extended to other domains facing similar challenges of multi-regime dynamics and rare event prediction. Aviation demand forecasting, urban transit planning, and multimodal transportation optimization all exhibit analogous patterns of routine operations punctuated by disruptive events, suggesting potential value from entropy-guided approaches.

Future research directions include several promising avenues: (1) extending the framework to multi-step forecasting through recursive prediction or direct multi-output architectures; (2) incorporating event calendars and real-time information feeds to enhance prediction for scheduled special events; (3) exploring non-monotonic gating functions and uncertainty-aware model fusion strategies; and (4) developing integration methods that could provide prediction intervals alongside point forecasts, enabling risk-aware operational planning during high-uncertainty periods identified by elevated entropy indicators.

## Figures and Tables

**Table 1:** Statutory Public Holidays in China and Corresponding Vacation Durations.

Attribute	Classification						
Name	Qingming	Labor Day	National Day	Dragon Boat	Mid-Autumn	New Year’s Day	Spring Festival
Number of Vacation Days	3	3~5	7~8	3	3	3	7~9

**Table 2:** Classification of Extreme Weather Events and Associated Impact Levels.

Attribute	Classification						
Name	Typhoon	Earthquake	Blizzard	Moderate to Heavy Snow	Heavy Rain	Freezing Rain	Strong Winds
Impact Level	High	High	High	Medium	Medium	Medium	Medium

**Table 3:** Parameter setting of the EA-ARIMA-Informer.

Category	Parameter	Value
Sample Entropy	Window length	30
	Embedding dimension	2
	Tolerance ratio	σ
Permutation Entropy	Order	3
	Delay	1
Transfer Entropy	Method	histogram-based and 10 bins
	Lag order	1
CEGF	Computation mode	ratio
	Threshold	90%
EA-ARIMA (low entropy)	Order	(2, 0, 1)
EA-ARIMA (high entropy)	Order	(1, 1, 2)
Informer	Encoder layers	5
	Hidden units	128
	Attention heads	4
	Head dimension	32
	Activation function	ReLU
	Learning rate	0.0001
	Optimizer	Adam

**Table 4:** Overall MAPE (%) of EA-ARIMA-Informer by City Level and Operational Scenario.

City Level	Overall	Workday	Holiday	Extreme
Large-scale City	4.39%	3.45%	6.33%	4.11%
Tier-1 City	4.26%	3.66%	5.43%	6.16%
Tier-2 City	5.08%	4.63%	7.42%	7.74%
Tier-3 City	7.82%	6.83%	9.76%	8.22%

**Table 5:** Performance of Baseline and Entropy-Augmented Models by City Level.

City Level	Method	MAPE	RMSE
Large-scale City	ARIMA	9.39%	47,879.56
	XGBOOST	6.20%	31,571.70
	Informer	6.43%	32,739.28
	EA-ARIMA	5.09%	23,516.37
	EA-Informer	4.79%	22,139.45
	EA-ARIMA-Informer	**4.39%**	**20,928.52**
Tier-1 City	ARIMA	6.46%	17,219.00
	XGBOOST	6.47%	17,247.93
	Informer	6.49%	16,027.18
	EA-ARIMA	4.57%	10,686.56
	EA-Informer	4.28%	11,049.47
	EA-ARIMA-Informer	**4.26%**	**10,701.42**
Tier-2 City	ARIMA	9.31%	6005.64
	XGBOOST	7.28%	5583.78
	Informer	7.58%	5633.06
	EA-ARIMA	5.68%	3727.54
	EA-Informer	5.48%	3364.10
	EA-ARIMA-Informer	**5.08%**	**3307.39**
Tier-3 City	ARIMA	15.95%	3532.29
	XGBOOST	13.75%	3389.74
	Informer	12.87%	3313.24
	EA-ARIMA	10.52%	2577.54
	EA-Informer	9.71%	2399.43
	EA-ARIMA-Informer	**7.82%**	**2121.18**

**Table 6:** Ablation Results under Holiday and Extreme-Weather Scenarios.

City Level	ARIMA-Informer		Feature-ARIMA-Informer		EA-ARIMA-Informer	
	MAPE	RMSE	MAPE	RMSE	MAPE	RMSE
Large-scale City	6.41%	44,193.56	5.79%	30,206.05	4.64%	27,954.60
Tier-1 City	6.45%	16,945.03	5.89%	16,989.30	5.85%	16,472.83
Tier-2 City	11.43%	4999.67	10.44%	5151.70	8.33%	5406.27
Tier-3 City	10.51%	3223.86	10.36%	3447.80	7.42%	3244.04

## Data Availability

The datasets presented in this article are not readily available due to privacy. Requests to access the datasets should be directed to the Railway Passenger Transport Department.

## References

[1] Wen K., Zhao G., He B., Ma J., Zhang H. (2022). A Decomposition-Based Forecasting Method with Transfer Learning for Railway Short-Term Passenger Flow in Holidays. Expert Syst. Appl..

[2] Li W., Sui L., Zhou M., Dong H. (2021). Short-Term Passenger Flow Forecast for Urban Rail Transit Based on Multi-Source Data. EURASIP J. Wirel. Commun. Netw..

[3] Ma M., Liu J., Cao J. (2014). Short-Term Forecasting of Railway Passenger Flow Based on Clustering of Booking Curves. Math. Probl. Eng..

[4] Zhang J., Yang Y., Yang L., Gao Z. (2026). Joint Short-Term Origin-Destination Demand Prediction for Multimodal Transport Systems. IEEE Trans. Pattern Anal. Mach. Intell..

[5] Lu Y., Wang J. (2025). Short-Term Passenger Flow Forecasting for Rail Transit Inte-Grating Multi-Scale Decomposition and Deep Attention Mechanism. Sustainability.

[6] Li D., Du S., Hou Y. (2024). Long-Term Passenger Flow Forecasting for Rail Transit Based on Complex Networks and Informer. Sensors.

[7] Du B., Peng H., Wang S., Bhuiyan M.Z.A., Wang L., Gong Q., Liu L., Li J. (2020). Deep Irregular Convolutional Residual LSTM for Urban Traffic Passenger Flows Prediction. IEEE Trans. Intell. Transp. Syst..

[8] Hao X., Zhong X., Yang J., Sun S., Zhang J. (2025). Multi-Task Learning-Based High-Speed Railway Inflow and Outflow Prediction with Large Language Models. Transp. Transp. Sci..

[9] Wei Z., Shan X. (2019). Research on Forecast Method of Railway Passenger Flow Demand in Pre-Sale Period. IOP Conf. Ser. Mater. Sci. Eng..

[10] Zhang G.P. (2003). Time Series Forecasting Using a Hybrid ARIMA and Neural Network Model. Neurocomputing.

[11] Yu S., Luo A., Wang X. (2023). Railway Passenger Flow Forecasting by Integrating Passenger Flow Relationship and Spatiotemporal Similarity. Intell. Autom. Soft Comput..

[12] Yang Y., Chen Z., Gao Y., Wang Z., Ding Z., Wu J. (2026). Network Traffic Forecasting with Transfer Learning-Based Algorithm for Long Continuous Missing Data. Expert Syst. Appl..

[13] Zhou S., Song C., Wang T., Pan X., Chang W., Yang L. (2022). A Short-Term Hybrid TCN-GRU Prediction Model of Bike-Sharing Demand Based on Travel Characteristics Mining. Entropy.

[14] Yang F., Shuai C., Qian Q., Wang W., He M., He M., Lee J. (2023). Predictability of Short-Term Passengers’ Origin and Destination Demands in Urban Rail Transit. Transportation.

[15] Wei T., Yang X., Xu G., Shi F. (2023). Medium-Term Forecast of Daily Passenger Volume of High Speed Railway Based on DLP-WNN. Railw. Sci..

[16] Tsai T.-H. (2014). A Self-Learning Advanced Booking Model for Railway Arrival Forecasting. Transp. Res. Part C Emerg. Technol..

[17] Shapovalova Y., Baştürk N., Eichler M. (2021). Multivariate Count Data Models for Time Series Forecasting. Entropy.

[18] Katambire V.N., Musabe R., Uwitonze A., Mukanyiligira D. (2023). Forecasting the Traffic Flow by Using ARIMA and LSTM Models: Case of Muhima Junction. Forecasting.

[19] Dou F., Jia L., Wang L., Xu J., Huang Y. (2014). Fuzzy Temporal Logic Based Railway Passenger Flow Forecast Model. Comput. Intell. Neurosci..

[20] Shi Z., Zhang N., Schonfeld P.M., Zhang J. (2020). Short-Term Metro Passenger Flow Forecasting Using Ensemble-Chaos Support Vector Regression. Transp. A Transp. Sci..

[21] Chuwang D.D., Chen W., Zhong M. (2023). Short-Term Urban Rail Transit Passenger Flow Forecasting Based on Fusion Model Methods Using Univariate Time Series. Appl. Soft Comput..

[22] Vivas E., Allende-Cid H., Salas R. (2020). A Systematic Review of Statistical and Machine Learning Methods for Electrical Power Forecasting with Reported MAPE Score. Entropy.

[23] Lu W., Zhang Y., Li P., Wang T. (2023). Mul-DesLSTM: An Integrative Multi-Time Granularity Deep Learning Prediction Method for Urban Rail Transit Short-Term Passenger Flow. Eng. Appl. Artif. Intell..

[24] He Y., Li L., Zhu X., Tsui K.L. (2022). Multi-Graph Convolutional-Recurrent Neural Network (MGC-RNN) for Short-Term Forecasting of Transit Passenger Flow. IEEE Trans. Intell. Transp. Syst..

[25] Deng S., Du J., Zhang J., Wang X. (2026). Deep Learning Approach for Short-Term Entry Passenger Flow Forecasting in Urban Rail Transit Stations. Eng. Appl. Artif. Intell..

[26] Ye J., Zhao J., Ye K., Xu C. (2022). How to Build a Graph-Based Deep Learning Architecture in Traffic Domain: A Survey. IEEE Trans. Intell. Transp. Syst..

[27] Wang Y., Zhang Z., Pi S., Zhang H., Pi J. (2025). Dual-Gated Graph Convolutional Recurrent Unit with Integrated Graph Learning (DG3L): A Novel Recurrent Network Architecture with Dynamic Graph Learning for Spatio-Temporal Predictions. Entropy.

[28] Lu W., Zhang Y., Vu H.L., Xu J., Li P. (2025). A Novel Integrative Prediction Framework for Metro Passenger Flow. J. Intell. Transp. Syst..

[29] Guan H., Dai Z., Guan S., Zhao A. (2019). A Neutrosophic Forecasting Model for Time Series Based on First-Order State and Information Entropy of High-Order Fluctuation. Entropy.

[30] Ponce-Flores M., Frausto-Solís J., Santamaría-Bonfil G., Pérez-Ortega J., González-Barbosa J.J. (2020). Time Series Complexities and Their Relationship to Forecasting Performance. Entropy.

[31] Bajić D., Japundžić-Žigon N. (2021). On Quantization Errors in Approximate and Sample Entropy. Entropy.

[32] Li Y., Fujita H., Li J., Liu C., Zhang Z. (2022). Tensor Approximate Entropy: An Entropy Measure for Sleep Scoring. Knowl.-Based Syst..

[33] Li G., Knoop V.L., Van Lint H. (2022). Estimate the Limit of Predictability in Short-Term Traffic Forecasting: An Entropy-Based Approach. Transp. Res. Part C Emerg. Technol..

[34] Ruiz Reina M.Á. (2021). Entropy Method for Decision-Making: Uncertainty Cycles in Tourism Demand. Entropy.

[35] Li J., Li W., Lian G. (2022). Optimal Aggregate Size of Traffic Sequence Data Based on Fuzzy Entropy and Mutual Information. Sustainability.

[36] Pan Y.A., Guo J., Chen Y., Cheng Q., Li W., Liu Y. (2024). A Fundamental Diagram Based Hybrid Framework for Traffic Flow Estimation and Prediction by Combining a Markovian Model with Deep Learning. Expert Syst. Appl..

[37] Zhang J., Mao S., Zhang S., Yin J., Yang L., Gao Z. (2025). EF-Former for Short-Term Passenger Flow Prediction during Large-Scale Events in Urban Rail Transit Systems. Inf. Fusion.

[38] Yang Y., Zhang J., Yang L., Yang Y., Li X., Gao Z. (2023). Short-Term Passenger Flow Prediction for Multi-Traffic Modes: A Transformer and Residual Network Based Multi-Task Learning Method. Inf. Sci..

[39] Tian Y., Wang D., Zhou G., Wang J., Zhao S., Ni Y. (2023). An Adaptive Hybrid Model for Wind Power Prediction Based on the IVMD-FE-Ad-Informer. Entropy.

[40] Ouedraogo I.A., Oki E. (2016). A Green and Robust Optimization Strategy for Energy Saving Against Traffic Uncertainty. IEEE J. Sel. Areas Commun..

[41] Qian W., Zhao Y., Zhang D., Chen B., Zheng K., Zhou X. (2024). Towards a Unified Understanding of Uncertainty Quantification in Traffic Flow Forecasting. IEEE Trans. Knowl. Data Eng..

[42] Zhang Y., Chen Y., Wang Z., Xin D. (2024). TMFO-AGGRU: A Graph Convolutional Gated Recurrent Network for Metro Passenger Flow Forecasting. IEEE Trans. Intell. Transp. Syst..

[43] Zou D., Wang S., Li X., Peng H., Wang Y., Liu C., Sheng K., Zhang B. MultiSPANS: A Multi-Range Spatial-Temporal Transformer Network for Traffic Forecast via Structural Entropy Optimization, 2023. Proceedings of the WSDM ’24: The 17th ACM International Conference on Web Search and Data Mining.

[44] Shannon C.E. (1948). A Mathematical Theory of Communication. Bell Syst. Tech. J..

[45] Richman J.S., Moorman J.R. (2000). Physiological Time-Series Analysis Using Approximate Entropy and Sample Entropy. Am. J. Physiol.-Heart Circ. Physiol..

[46] Bandt C., Pompe B. (2002). Permutation Entropy: A Natural Complexity Measure for Time Series. Phys. Rev. Lett..

[47] Schreiber T. (2000). Measuring Information Transfer. Phys. Rev. Lett..

[48] Wulff S.S. (2017). Time Series Analysis: Forecasting and Control, 5th Edition. J. Qual. Technol..

[49] Zhou H., Zhang S., Peng J., Zhang S., Li J., Xiong H., Zhang W. Informer: Beyond Efficient Transformer for Long Sequence Time-Series Forecasting. Proceedings of the Thirty-Fifth AAAI Conference on Artificial Intelligence.

